# Type A thymoma with simultaneous solitary intrapulmonary metastasis: A case report

**DOI:** 10.1111/1759-7714.13975

**Published:** 2021-05-07

**Authors:** Tsutomu Tatematsu, Katsuhiro Okuda, Katsuhiko Endo, Hideo Hattori, Takuya Matsui, Risa Oda, Tadashi Sakane, Keisuke Yokota, Ryoichi Nakanishi

**Affiliations:** ^1^ Department of Oncology, Immunology and Surgery Nagoya City University Graduate School of Medical Sciences Nagoya Japan; ^2^ Department of Surgery Nagoya Memorial Hospital Nagoya Japan; ^3^ Department of Pathology and Molecular Diagnostics Nagoya City University Graduate School of Medical Sciences Nagoya Japan

**Keywords:** pulmonary metastasis, thymoma, type A

## Abstract

A 79‐year‐old woman was referred to our facility because of an abnormal chest shadow. Chest computed tomography (CT) showed a solitary right middle lung nodule with a maximum diameter of 3 mm and anterior mediastinal nodule with a maximum diameter of 21 mm. The lung nodule was suspected of being a primary lung cancer rather than a metastatic tumor because there were no primary malignant tumors, apart from an anterior mediastinal tumor visible on diagnostic imaging, including F^18^ fluorodeoxyglucose‐positron emission tomography, and a solitary lung nodule. Partial lung resection by video‐assisted thoracoscopic surgery (VATS) was performed, and the intraoperative frozen section of the tumor tissue resulted in a diagnosis of carcinoid tumor. As a result, right middle lobectomy by VATS was performed. The final histological diagnosis of the permanent specimen was intrapulmonary type A thymoma. VATS thymectomy was performed three months later. The histological diagnosis was type A thymoma with intrapulmonary metastasis (Masaoka stage IVb). Additional therapy was not performed because complete resection was achieved. Follow‐up CT was performed once every six months after the operation. The patient has been followed up for one year without any further recurrence.

## Graphical abstract

It is rare for type A thymoma to have solitary and tiny pulmonary metastasis. Intrapulmonary metastasis of thymomas should be kept in mind in the differential diagnosis of small pulmonary nodules, even when they are solitary.

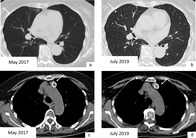

## INTRODUCTION

Type A thymoma is a relatively rare histotype that progresses very slowly. The distant metastasis of type A thymomas is rare. We herein report a case of type A thymoma with solitary pulmonary metastasis.

## CASE REPORT

**FIGURE 1:**
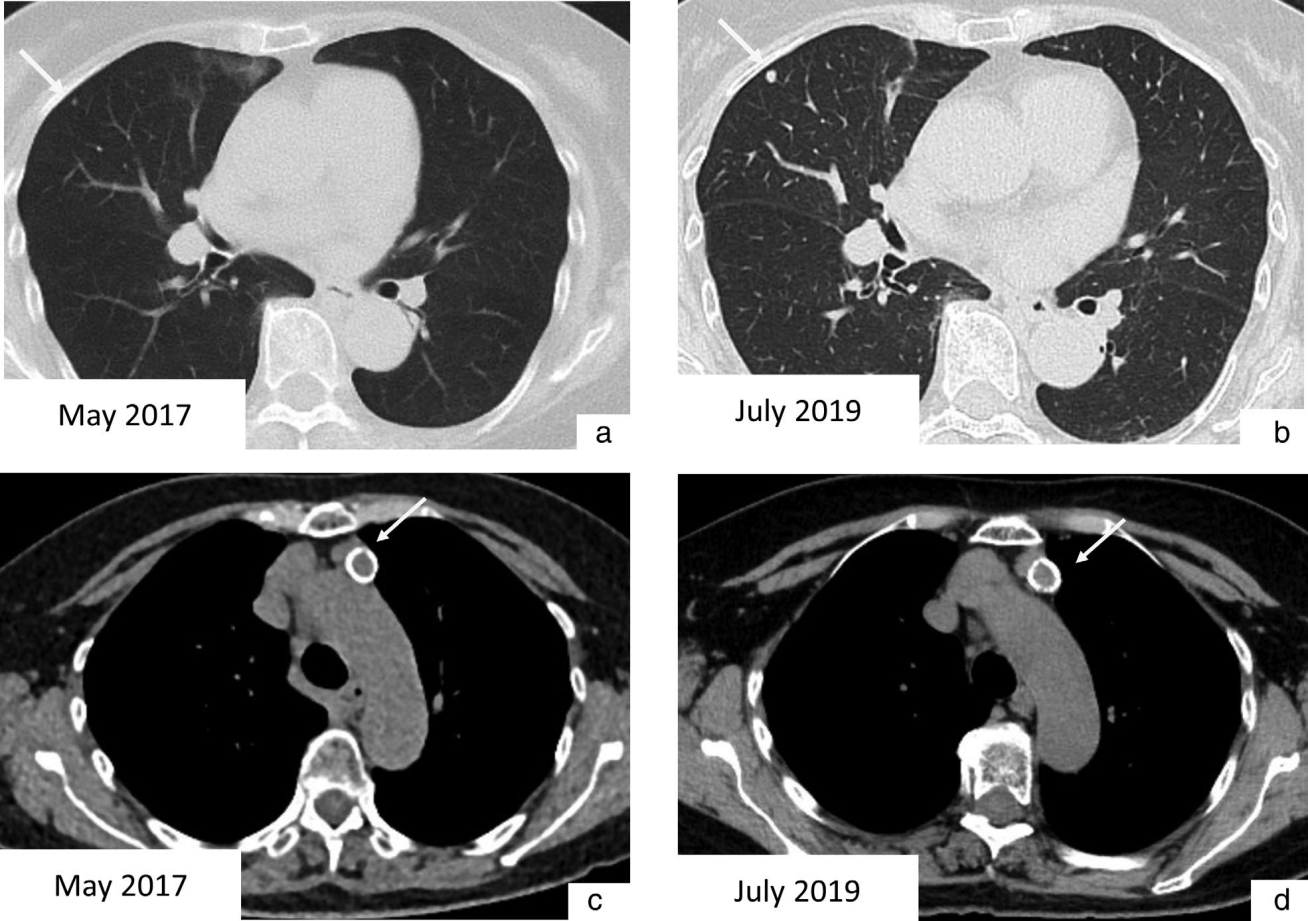
(a) A pulmonary nodule in the right middle lobe was detected by chest computed tomography (CT). (b) It had grown slightly over a two‐year period. (c) An anterior mediastinal tumor with ring‐shaped calcification was detected by chest CT. (d) It had not changed markedly over a two‐year period

**FIGURE 2:**
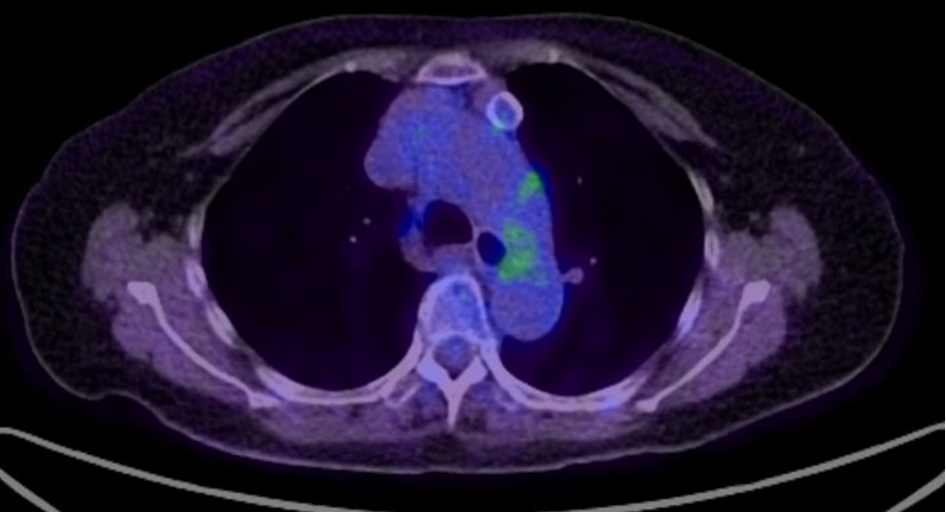
F^18^ fluorodeoxyglucose‐positron emission tomography (F^18^ FDG‐PET) showed no uptake of FDG in the anterior mediastinal tumor

A 79‐year‐old woman was referred to our hospital because of an abnormal chest shadow on chest computed tomography (CT). She had never smoked and had no family history of cancer. CT scan showed a small nodule (maximum diameter: 3 mm) in the right middle lobe of the lung and an anterior mediastinal nodule (major axis: 21 mm) with ring‐shaped calcification (Figure 1(b), (d)). These nodules had been noted on the CT performed two years earlier, and the pulmonary nodule had grown slightly during the two‐year interim (Figure 1(a), (b)), although the anterior mediastinal nodule had not grown significantly (Figure 1(c), (d)). F^18^ fluorodeoxyglucose‐positron emission tomography (F^18^ FDG‐PET) showed no uptake of FDG in either the lung or anterior mediastinal nodule (Figure 2). Blood tests only revealed elevated alpha fetoprotein (AFP) levels, at 14.1 ng/ml (normal: ≦10.0 ng/ml), with no other tumor markers or antiacetylcholine receptor (AChR) antibody noted.

The lung nodule was suspected of being a primary lung cancer because it was solitary and there were no other suspected primary malignant tumors. The anterior mediastinal nodule was suspected of being a vascular malformation because the nodule showed ring‐shaped calcification and no uptake of FDG on positron emission tomography‐computed tomography (PET‐CT).

**FIGURE 3:**
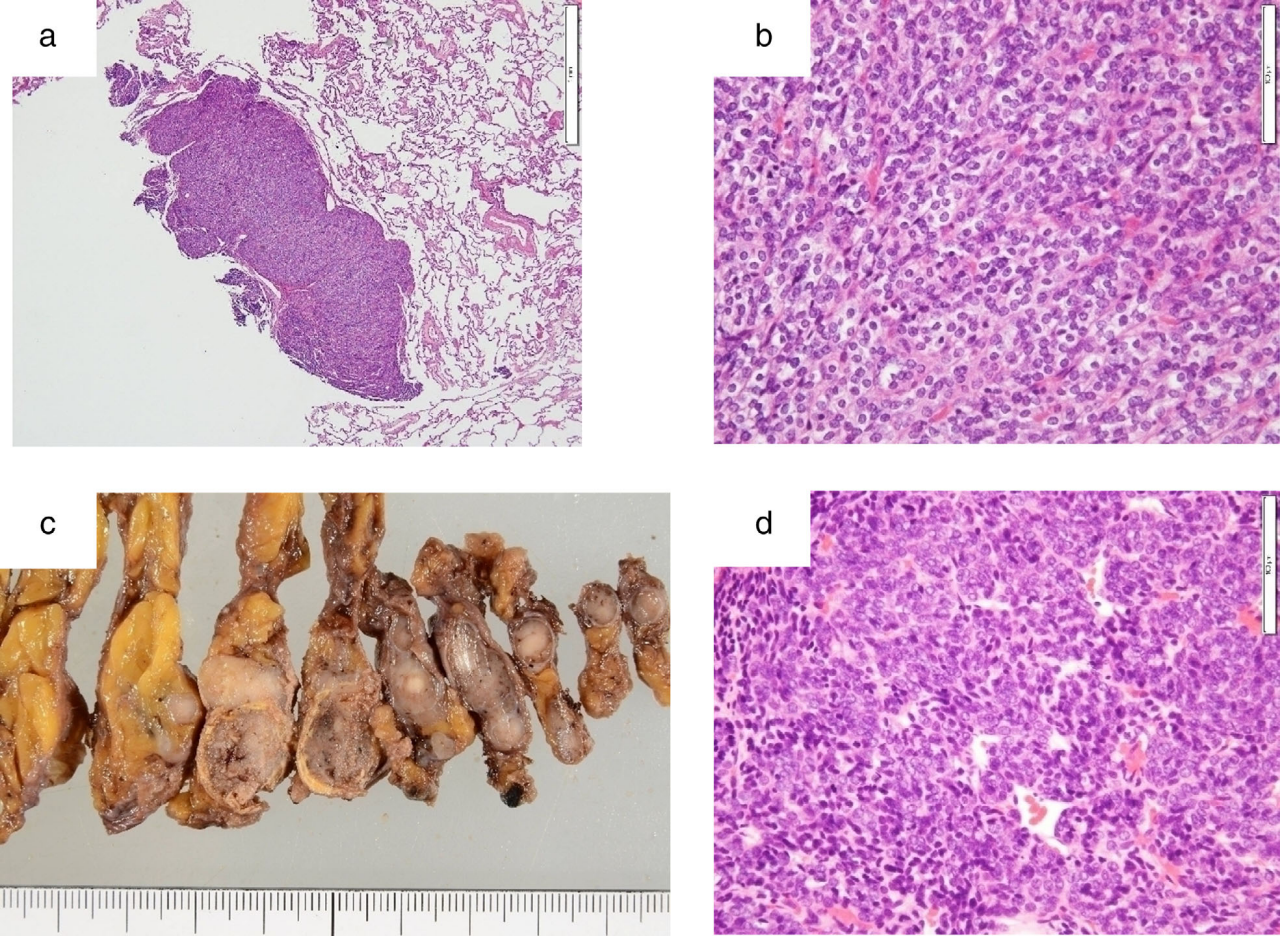
The microscopic findings of the resected intrapulmonary tumor. (a) A well‐defined tumor had formed in the pulmonary region according to the microscopic findings (hematoxylin and eosin [H&E] staining, original magnification x20). (b) The small tumor cells grew like alveolar cells (H&E staining, original magnification x400). The macro‐ and microscopic findings of the resected anterior mediastinal tumor. (c) A well‐defined tumor with ring‐shaped calcification had formed in the thymus. (d) Spindle‐shaped tumor cells were growing in the thymus (H&E staining, original magnification x400)

Partial lung resection by video‐assisted thoracoscopic surgery (VATS) was performed, and a carcinoid tumor was diagnosed by using an intraoperative frozen section. Therefore, additional resection (right middle lobectomy) was performed. However, the final pathological findings revealed a well‐defined tumor, and the small tumor cells grew like alveolar cells on hematoxylin and eosin staining (Figure 3(a)). Furthermore, the cells did not show carcinoid characteristics on immunostaining. The tumor was therefore diagnosed as intrapulmonary metastatic tumor of type A thymoma (Figure 3(b)). Because the mediastinal nodule was suspected of being thymoma, VATS thymectomy was performed three months later (Figure 3(c)). The histological diagnosis of the anterior mediastinal tumor was type A thymoma (Masaoka stage IVb) (Figure 3(d)). Additional therapy was not performed because the tumor had been completely resected. Follow‐up CT was performed every six months after the operation. The patient has been followed for one year without any further recurrence.

## DISCUSSION

Thymomas are anterior mediastinal tumors with low to moderate malignancy. In the database from the Latin‐American Consortium for the Investigation of Lung Cancer (CLICaP), the five‐year OS rates were 73.4% for stage I–II, 63.8% for III–IVa and 51% for IVb.^1^ The World Health Organization (WHO) classification system subdivides thymomas into type A, AB, B1, B2, and B3.^2^ Among these types, type A thymoma has the lowest risk of malignancy and a good prognosis.^3^ In a previous study that analyzed 4221 thymomas from the International Thymic Malignancy Interest Group database, type A thymomas with stage I or II accounted for 90% of all type A thymomas and stage IV was rare.^4^

The most common site for metastasis or recurrence in thymomas is pleural dissemination. It is quite rare for thymomas to cause a single pulmonary metastasis as in this case.^5^, ^6^ Khandelwal et al. analyzed the data of 62 patients with thymomas and reported that 14 (22.5%) cases had metastasis and recurrence and only one of those 14 cases had lung metastasis.^6^ In contrast, several cases of type A thymoma with lung metastasis have previously been reported.^6^, ^7^, ^8^, ^9^ For example, Vladislav et al. reported that six (26%) of 23 patients with type A thymoma had lung metastasis.^7^

Some type A thymomas are extremely progressive, and atypical type A thymoma was added to thymomas subtype in the 2015 WHO classification.^2^, ^6^ The pathological characteristic of atypical type A thymomas include hypercellularity, increased mitotic activity, and necrosis. These characteristics may thus correlate with an advanced stage and poor prognosis.^2^, ^6^, ^7^, ^9^, ^10^

In the present case, the thymoma and intrapulmonary metastasis of the thymoma were found at the same time. The simultaneous solitary intrapulmonary metastasis of thymoma, not recurrence, is quite rare. It was difficult to diagnose the lesion as a thymoma and solitary lung metastasis of thymoma by diagnostic imaging. The pulmonary nodule was not suspected of being a metastatic lung tumor because it was solitary and tiny, and there were no lesions, including the anterior mediastinal tumor that showed the uptake of FDG on PET. The anterior mediastinal tumor was not considered a malignant tumor based on the PET finding until the pathological results of the pulmonary nodule were obtained. The thymoma was conventional type A because it did not have pathological characteristics of atypical type A.

In conclusion, it is necessary to keep lung metastasis of thymoma in mind as a differential diagnosis of a solitary lung nodule.

## CONFLICT OF INTEREST

The authors have no conflicts of interest to declare.
